# Paleomagnetism of IODP Site U1380: Implications for the Forearc Deformation in the Costa Rican Erosive Convergent Margin

**DOI:** 10.1038/s41598-018-29243-7

**Published:** 2018-07-30

**Authors:** Yong-Xiang Li, Xixi Zhao, Siyi Xie, Luigi Jovane, Katerina Petronotis

**Affiliations:** 1 State Key Laboratory for Mineral Deposits Research, School of Earth Sciences and Engineering, Institute of Geophysics and Geodynamics, Nanjing University, Nanjing, 210046 China; 2 State Key Laboratory of Marine Geology, Tongji University, Shanghai, 200092 China; 3 Instituto Oceanográfico, Universidade de São Paulo, São Paulo, SP 05508 Brazil; 4 International Ocean Discovery Program, Texas A&M University, College Station, Texas 77845 USA

## Abstract

The destructive nature of subduction erosion poses challenges to fully understanding the evolution of erosive convergent margins that are critical to understanding crustal recycling and seismogenesis. Forearc deformation holds important clues to the evolution of erosive convergent margins. Here we present detailed paleomagnetic and structural analyses of IODP Site U1380 cores from the middle slope of the forearc of the Costa Rican erosive convergent margin. The analyses reveal a strong deformation zone from ~490 to ~550 mbsf that is characterized by abundant fissility/foliations shallower than the bedding. Similar relatively strong deformation zones are recognized from the frontal prism and upper slope sites, and are broadly correlative, forming a zone of strong deformation across the forearc. This zone spans ~2.0 to 1.83 Ma and the deformation likely occurred briefly at ~1.80 Ma. The widespread, short-lived, and strong deformation is interpreted as a result of intense subhorizontal shear following the rapid forearc subsidence driven by the dramatic subduction erosion associated with the abrupt onset of the Cocos Ridge subduction. Given the typical occurrence of forearc subsidence by subduction erosion, similar styles of deformation are probably common in other erosive convergent margins as well.

## Introduction

Convergent plate boundaries are considered either accretional or erosive on the basis of whether material is accreted onto or eroded away from the overriding plate in subduction zones^1–3^. About half of the worldwide convergent margins are erosive^2,3^. Erosive convergent margins are characterized by subduction erosion at the frontal and/or basal part of forearc wedges^4–7^ and thus important for crustal material transfer. Erosive convergent margins are also the places where large earthquakes occur^8^. Therefore, elucidating the evolution of erosive convergent margins are critical in understanding crustal recycling^9^ and origin of earthquakes^10^ in subduction zones. However, the destructive nature of subduction erosion poses significant challenges in deciphering the rock record at erosive convergent margins. Nevertheless, forearc wedges in erosive convergent margins are situated at the forefront in the subduction process and their deformation histories hold important clues to the evolution of this type of convergent margin.

**Figure 1:**
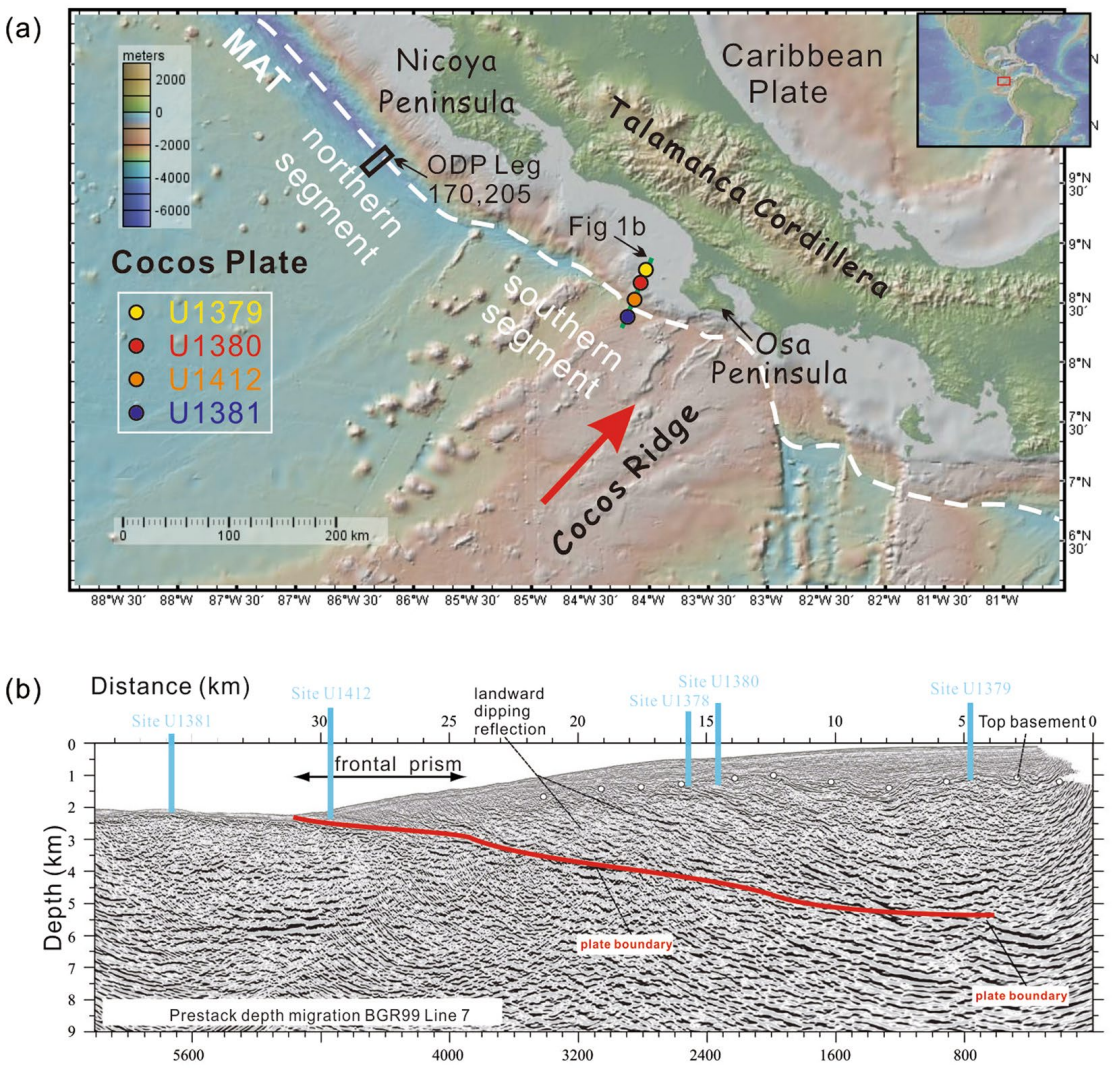
Tectonic setting and locations of ODP Legs 170 and 205 as well as IODP Expedition 334 and 344 drill sites on the Costa Rican convergent margin (GeoMapApp version 3.3.9, http://www.geomapapp.org). (**a**) Location of IODP sites along the BGR99-Line 7 transect. (**b**) Seismic profile of the BGR99-Line 7 transect showing the locations of IODP sites at the Cocos Ridge and different parts of the forearc (modified after^8^). MAT, Middle American Trench. The red arrow indicates the direction of the Cocos Ridge subduction.

The western margin of Costa Rica in Central America is a typical erosive convergent margin where the Cocos plate subducts beneath the Caribbean plate (Fig. 1). The Costa Rican convergent margin can be broadly divided into the northern and southern segments^11^ (Fig. 1). The oceanic crust in the northern segment is relatively older and the ocean floor morphology is largely smooth. Ocean drilling program (ODP) Legs 170 and 205 investigated subduction fluxes and fluid flow at the northern segment of the margin^12,13^. Drilling of ODP Legs 170 and 205 penetrated the frontal prism of the overriding sedimentary wedge and investigated the forearc tectonic processes at this part of the margin involving subduction erosion, sediment off-scraping, and underplating^6,14,15^.

The southern segment of the Costa Rican margin is drastically different from the northern counterpart. Here, the Cocos Ridge (CR), an overthickened aseismic ridge^16,17^ with a significantly rough surface^18^, subducts perpendicular to the Middle American Trench (MAT) at ~9.0 cm/yr^19,20^ at a shallow angle (<10°)^21^ beneath the Caribbean plate (Fig. 1). Subduction erosion in the southern segment is much stronger than in the northern segment and the landward embayment of the MAT directly inboard of the CR presents the most diagnostic geomorphological expression of subduction erosion at this margin^22^ (Fig. 1a). The CR subduction has also caused strong and widespread upper plate deformation^23–27^, and produces frequent and large earthquakes^8,21,28,29^. Integrated Ocean Drilling Program (IODP) Expeditions 334 and 344 cored not only the frontal prism (Site U1412), but also the subducting CR (Sites U1381 and U1414) and middle slope (Sites U1378 and U1380), and the upper slope (Sites U1379 and U1413) offshore southern Costa Rica (near Osa Peninsula)^8,29^ (Fig. 1b, BGR99-Line7), providing a unique opportunity to examine the forearc deformation history. Sedimentary facies analysis shows that a layer of shell fragment–bearing nearshore facies of coarse sandstones sandwiched into deep-water environment facies, suggesting a km-scale uplift followed by a rapid subsidence of the forearc^7^. The vertical displacement was attributed to the onset of CR subduction that may have also caused a hiatus in the sediments on the subducting CR^30,31^. Also, in contrast to the sediment-starved trench, sediment accumulation in the forearc basin is high, indicating that the forearc is depositionary and subduction erosion appears to be compensated by the deposition of terrigenous sediments in the forearc basin^32^. Although sediments in the upper slope appear to exhibit different styles of deformation before and after the deposition of the nearshore facies^32^, it is not clear whether the difference in deformation style is pervasive in the forearc largely because the structural architecture in the forearc has generally not been examined in detail. In addition, although nannofossil and foramineral data provide an important chronological framework for these sites, the lack of a detailed chronology impedes our understanding of the deformation history and the sedimentary evolution for the forearc basin.

In this paper, we examine the deformation style and history at the middle slope Site U1380 in the southern segment of the Costa Rican margin. The deformation style is investigated using the anisotropy of magnetic susceptibility (AMS) together with the structural data collected during the expeditions^8,29^. In addition, a detailed paleomagnetic study refines the chronological framework and constrains the deformation history at the middle slope site. Furthermore, a comparative analysis of the deformation in the middle slope, the frontal prism, and the upper slope allows an improved understanding of the forearc deformation of the Costa Rican erosive convergent margin.

## Results

### Anisotropy of magnetic susceptibility (AMS) of Hole U1380C

**Figure 2:**
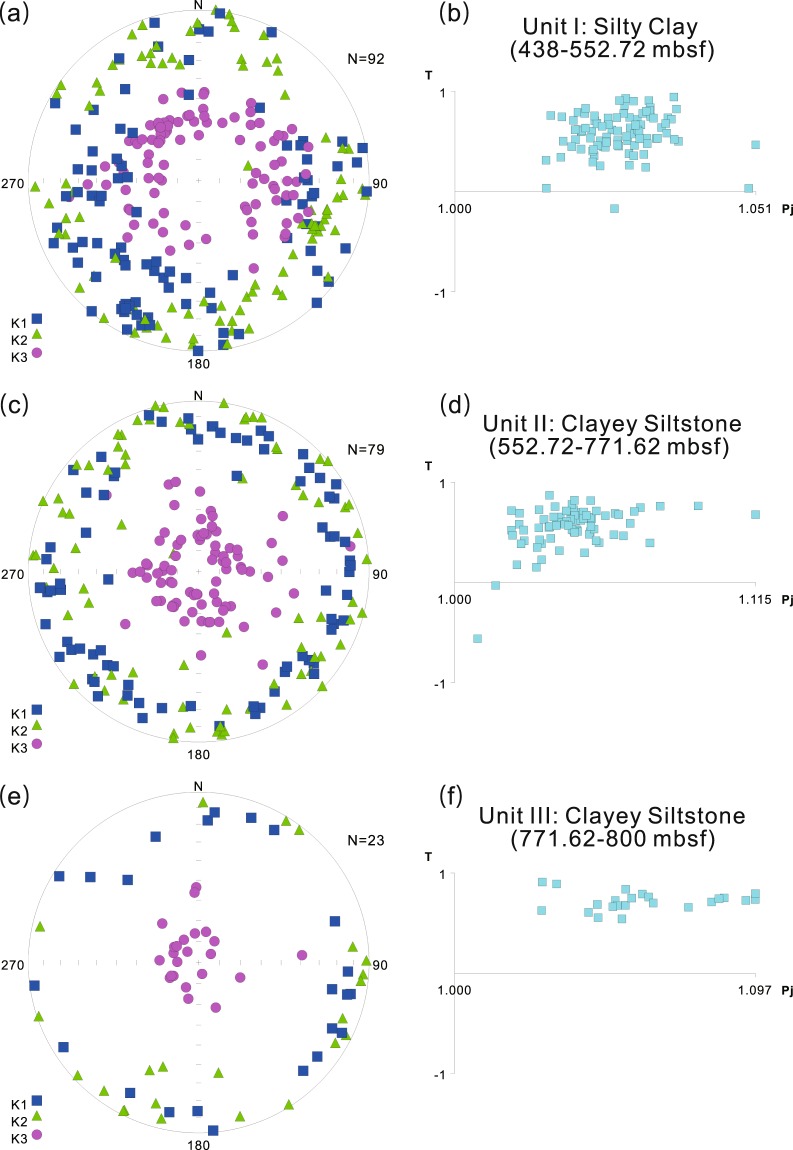
Anisotropy of magnetic susceptibility (AMS) data for the three lithological units of Hole U1380C. (**a**,**c**,**e**) Equal-area projections of principal axes in the geographic coordinates; N, the number of samples; K_1_, K_2_, K_3_ are the maximum, intermediate, and minimum axes of the AMS ellipsoid. (**b**,**d**,**f**) Shape and degree of anisotropy of the ellipsoid; T, shape factor, T = [0, 1], oblate; T = [−1, 0], prolate; Pj, degree of anisotropy^33^.

Hole U1380C sediments comprise three lithological units including, from bottom to top, silty claystone (Unit III), clayey siltstone and sandstone (Unit II), and silty clay (Unit I). Samples from all three units show predominantly oblate fabrics^33^ (Fig. 2). While all the K_max_ and K_int_ axes are horizontal or subhorizontal, the K_min_ axes appear to show a change from the vertical to subvertical from Unit III, through Unit II, to Unit I (Fig. 2a,c,e). For Unit III, the K_min_ axes are nearly vertical. The K_min_ axes of Unit II cluster around the vertical with a mean of ~65.8° ± 3.7°. For Unit I, the K_min_ axes are spread out around the vertical with a mean of ~54.1° ± 2.6°. The degree of anisotropy ranges from ~1.03 to ~1.1 for Unit III, mainly from 1.02 to 1.06 for Unit II, and mostly from 1.02 to 1.03 for Unit I (Fig. 2b,d,f).

It is well known that deposition of sediments in still water can produce oblate fabrics that are characterized by K_min_ perpendicular to the horizontal bedding as gravity is the predominant force^34^. The K_min_ tilt off the vertical could be depositional or tectonic related (tilted beds) in origin. Sediment deposition on a slope, or in the presence of current^34,35^, or grain collision or viscous suspension during sediment settling^36^ can lead to K_min_ tilt off the vertical. K_min_ tilt of the oblate AMS fabrics of the deep sea sediments from the Nankai Trough accretionary prism has been employed to investigate the sediment transport/deposition processes^37^ and the degree of deformation in the Nankai Trough accretionary prism^38^. Similarly, the K_min_ tilt of AMS fabrics in the upper hemipelagic/pelagic sediments on the subducting Cocos plate of the Costa Rican convergent margin is believed to be associated with the initial underplating of sediments beneath the margin^15^. However, the K_min_ tilt at the base of the hemipelagic unit was likely related to the tilted strata because steep bedding dip was observed^14^.

**Figure 3:**
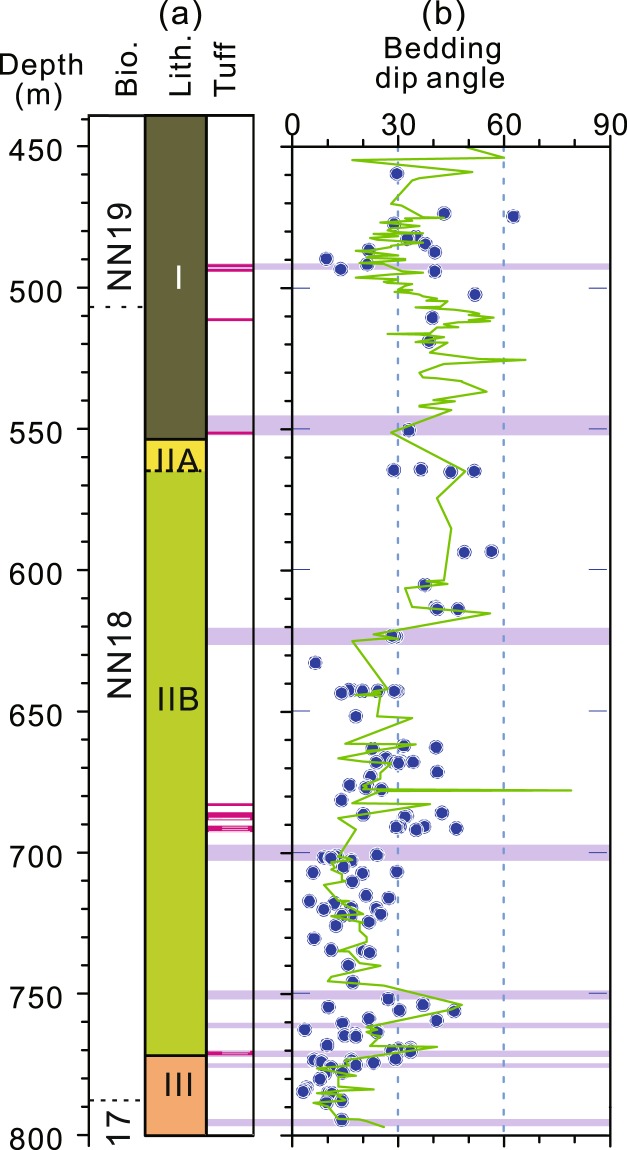
Downcore variations in the AMS K_min_ tilt and bedding dip measurements for Hole U1380C. (**a**) Bio., nannofossil zones; lith., Hole U1380C lithologic units; Tuff, ash layers indicated by pink lines. (**b**) Blue dots denote bedding dip angles measured by shipboard structural geologists^8^ and the green curve represents the degree of tilt of the AMS K_min_ axes off the vertical, a proxy for bedding dip. The purple bands represent the breccia/fracture zones. Depth is in meters below seafloor (mbsf).

At Site U1380, inclined bedding of the cores was observed during IODP Expedition 344^8^. The dip of the inclined bedding was measured and documented by the shipboard structural geologists during the expedition. This provides an excellent opportunity to examine the origin of the K_min_ tilt. Comparison of the measured dips of the inclined bedding with the K_min_ tilt of the AMS data shows that these two parameters are broadly consistent (Fig. 3). Although the depth levels of these datasets are not exactly one-to-one correlated due to the fact that the two datasets were collected independently, the K_min_ tilt and the bedding dip of the proximal depth levels are, by and large, similar with differences mostly within 10° (some within 5°, Table S1). The generally minor difference could be explained by the combined uncertainty in measurements of the two parameters and the minor difference in the proximal depths. In essence, K_min_ tilt is of tectonic origin and overall tracks bedding dip at this site. Therefore, K_min_ tilt can be used as a proxy for the steepness of tilted strata at Site U1380.

### Paleomagnetic results of Hole U1380C

**Figure 4:**
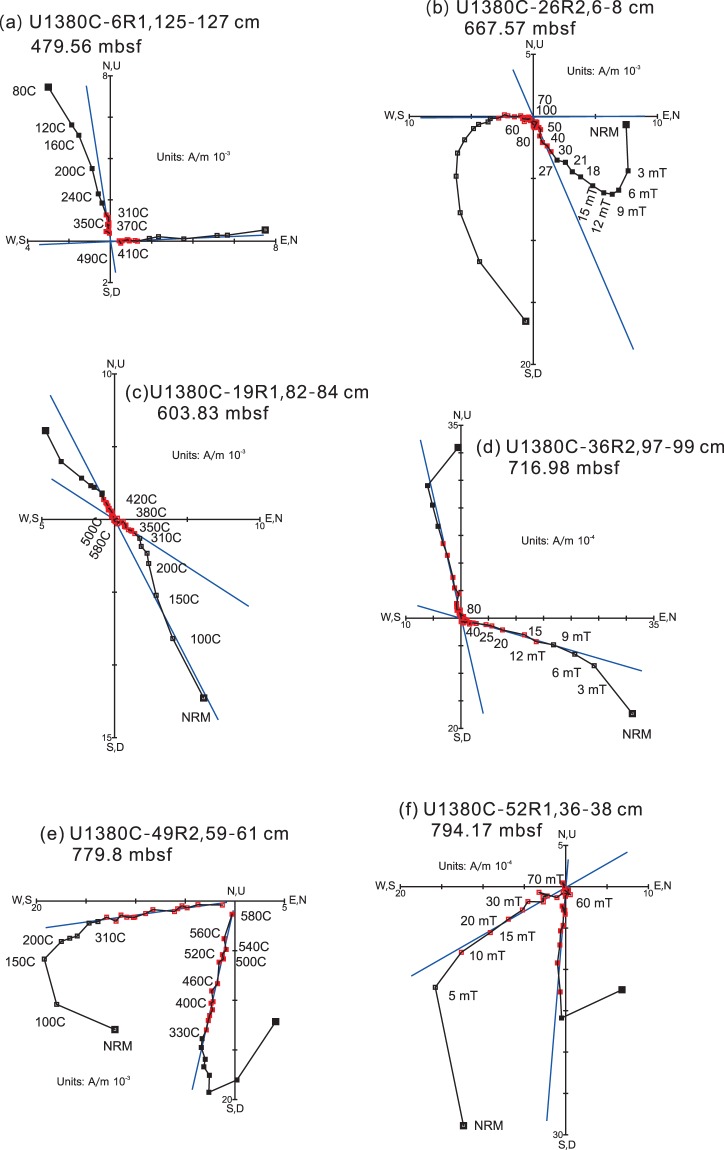
Representative thermal and AF demagnetization results for Hole U1380C samples. Solid squares indicate the horizontal magnetization components, and open squares denote the vertical components. The blue straight lines represent the characteristic remanent magnetization component determined using principal component analysis^45^. Sample depths are given as meters below seafloor (mbsf).

Natural remanent magnetization (NRM) of the samples ranges from 3.10 × 10^−4^ to 0.12 A/m with a mean of 8.78 × 10^−3^ A/m. The high NRM intensity values correspond to intervals near ash layers. Demagnetization results show that nearly half of the specimens (49 out of 109) from Unit I display erratic demagnetization trajectories. For the remaining 60 specimens, AF demagnetization, typically from ~20 to 40 mT, or thermal demagnetization, typically from 280 °C to 500 °C, effectively isolated the characteristic remanent magnetization (ChRM) (Fig. 4a). Samples from Units II and III generally yield two-component magnetizations with the demagnetization trajectory decaying toward the origin. The low coercivity (or low temperature) component can be removed by ~15 mT or 200 °C and the ChRMs are usually isolated between ~20 mT and ~80 mT or between ~310 °C and ~580 °C (Fig. 4b–f). Thermal demagnetization data also show that either the remanence is completely cleaned by 600 °C or a rapid decay in intensity occurs between ~550° and 600 °C, suggesting that the remanence is carried by magnetite. Detailed rock magnetic investigation of Hole U1380C samples shows that pseudo-single domain (PSD) magnetite is the dominant magnetic mineral in Hole U1380C^39^.

We use the following criteria to select the ChRMs. First, ChRMs with a maximum angular deviation (MAD) greater than 16° are deemed unreliable and are excluded from further analysis. Second, given that drilling can induce a near-vertical overprint^40,41^ and the site was located at a low latitude region^42^, ChRMs with inclinations steeper than 70° may be affected by drilling-induced remagnetization^40,41^ and are rejected. Third, since the cores were drilled with the rotary core barrel (RCB) and were not fully oriented, we only use downhole changes in inclination to construct the magnetostratigraphy. The ChRMs data are summarized in Table S2.

**Figure 5:**
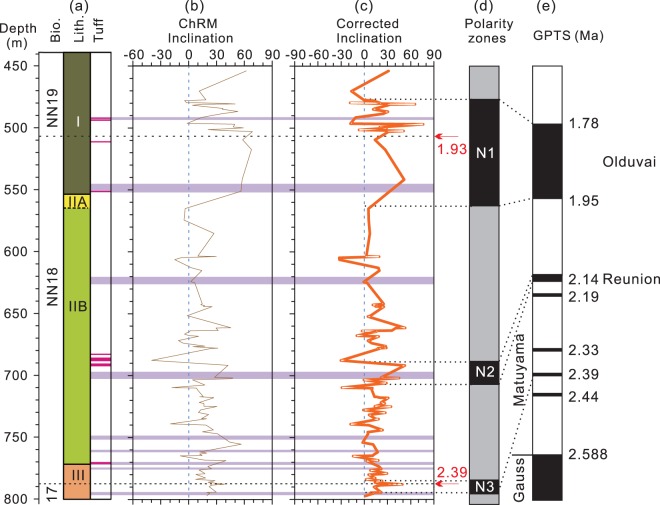
Paleomagnetic data for Hole U1380C. (**a**) Bio., nannofossil zones; lith., Hole U1380C lithologic units; (**b**) Inclinations of the characteristic remanent magnetization (ChRM) defined using principal component analysis. (**c**) Inclinations corrected for tilted beds; the red arrows mark the biostratigraphic age boundaries (Ma). (**d**) Magnetozones tentatively defined based on the corrected inclinations and the biostratigraphic constraint. (**e**) The Geomagnetic Polarity Time Scale (GPTS) of the early Quaternary. Color codes and depth are the same as those in Fig. 3.

Because beds were tilted and K_min_ tilt approximates the steepness of tilted strata, tilt correction was performed by restoring K_min_ axes to the vertical. An inclination-only fold test shows that k_s_/k_g_ = 1.33, where k_s_ and k_g_ are the precision parameters in stratigraphic and geographic coordinates respectively, suggesting that the grouping of inclinations is improved after tilt correction. Therefore, the ChRM is likely primary in origin and the tilt-corrected inclination can be used to establish magnetostratigraphy. The assignment of magnetozones was aided by the biostratigraphic results from this core. Biostratigraphic data show that the nannofossil Zone NN18 is well defined with its upper boundary at ~505 mbsf and the lower boundary at ~790 mbsf, respectively^8^. The upper and lower boundary of Zone NN18 corresponds to 1.93 Ma and 2.39 Ma, respectively^8^. With this constraint, the predominant positive inclination values between ~470 mbsf and ~560 mbsf (N1) are assigned to a normal polarity zone corresponding to the Subchron C2n (Olduvai), which ranges from 1.778 to 1.945 Ma^43^ (Fig. 5). Accordingly, the positive inclination values around 790 mbsf are assigned to a short normal polarity (N3) corresponding to the brief subchron at ~2.39 Ma^43^.

## Discussion

Faults and fractures occur frequently throughout Hole U1380C. One prominent structural feature is the occurrence of a broad shear zone between ~490 and ~550 mbsf ^8^. This shear zone occurs in the lowermost part of Unit I, which consists mainly of silty clays with sandstones, and right above Subunit IIA, which consists of sandstones with shell fragments and represents shallow water facies. This shear zone is characterized by abundant faults, fractures, and deformation bands, as well as elevated fluid pressure^8^. Abundant fissility/foliation was observed in the shear zone^8^.

**Figure 6:**
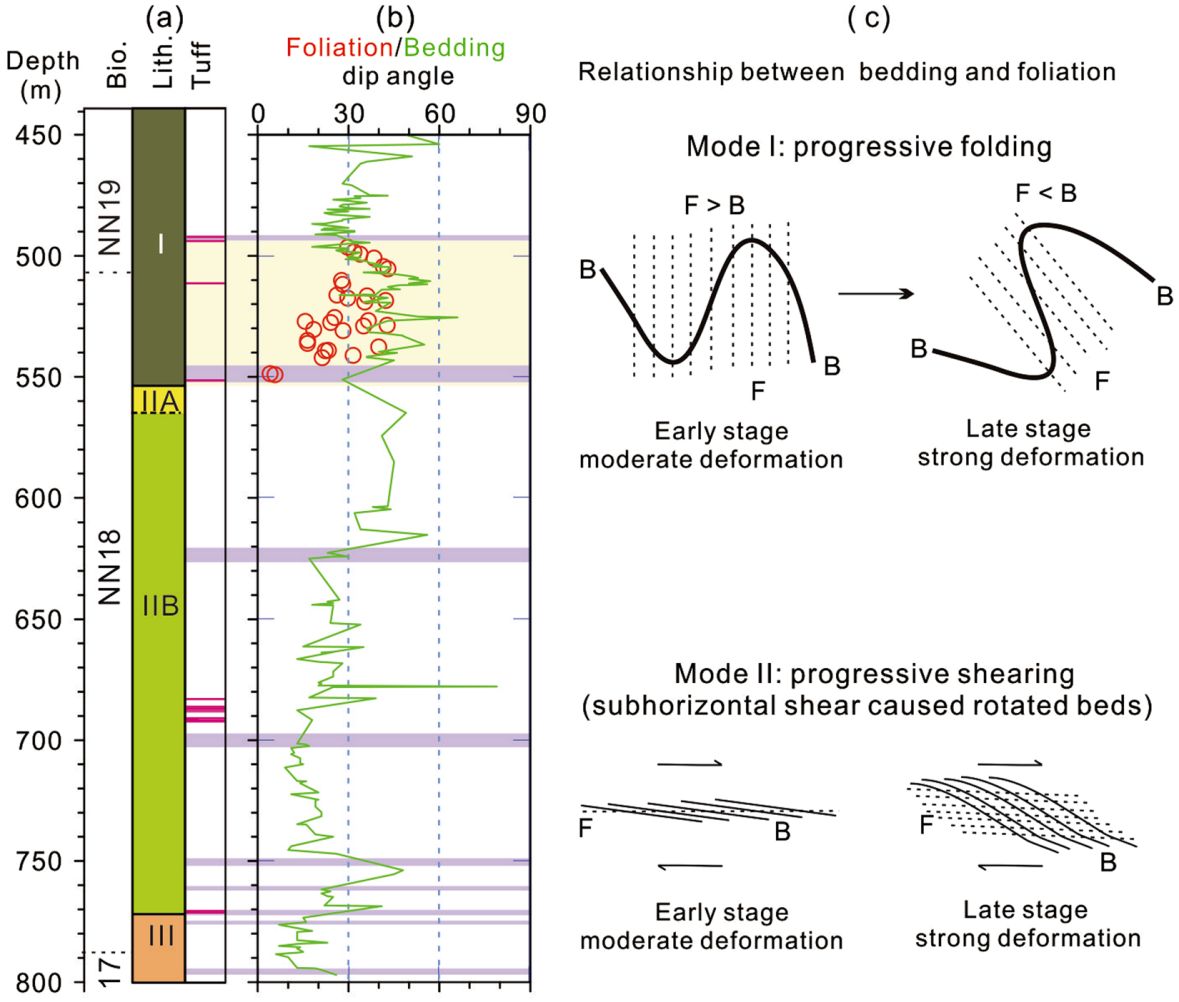
The relatively strong deformation zone (light yellow band) recognized in Hole U1380C. (**a**) Bio., nannofossil zones; lith., Hole U1380C lithologic units; (**b**) Red circles denote fissility/foliation^8^ and the green curve represents the bedding; The fissility/foliation is generally shallower than the bedding between ~490 and 550 mbsf, demarcating the relatively strong deformation zone. (**c**) Illustration of the relationship between bedding and foliation in two modes of deformation: Mode I, progressive folding; Mode II, progressive shearing. F, foliation; B, bedding; F > B, foliation is steeper than bedding; F < B, foliation is shallower than bedding. Color codes and depth are the same as those in Fig. 3.

The bedding dips are variable, but are generally <30° below ~630 mbsf and >30° above 630 mbsf (Fig. 3b). Within the broad shear zone between ~490 and 550 mbsf, the bedding dip, as represented by the degree of tilting off the vertical of the K_min_ axes of the magnetic fabrics (Fig. 3b), is generally steeper than the dip of fissility/foliation (Fig. 6b). Indeed, shipboard inspection of the cores confirms that the fissility/foliation is shallower than the bedding in the broad shear zone^8^. The occurrence of such a relationship between fissility/foliation and bedding is interesting and indicates a zone of relatively strong deformation at this site. One way to produce such a relationship is by progressive folding (Fig. 6c, Mode I). Since fissility/foliation is developed parallel to the axial plane of a fold in a compressional regime, progressive folding eventually leads to an overturned limb of the fold at a late stage of deformation when fissility/foliation becomes shallower than the bedding (Fig. 6c, Mode I). However, lithological and structural analyses show no overturned beds in the cores, suggesting that progressive folding may not be the cause. Another way to produce such a relationship is by a sub-horizontal simple shear (Fig. 6c, Mode II). In the initial stage, a simple shear probably occurred sub-parallel to the bedding. As the sub-horizontal simple shear continued, beds were progressively rotated (tilted) so that bedding became steeper than fissility/foliation (Fig. 6c, Mode II). In essence, the observation that bedding is steeper than foliation in the shear zone at ~490–550 mbsf is significant and provides evidence for a zone of relatively strong deformation in the cores.

**Figure 7:**
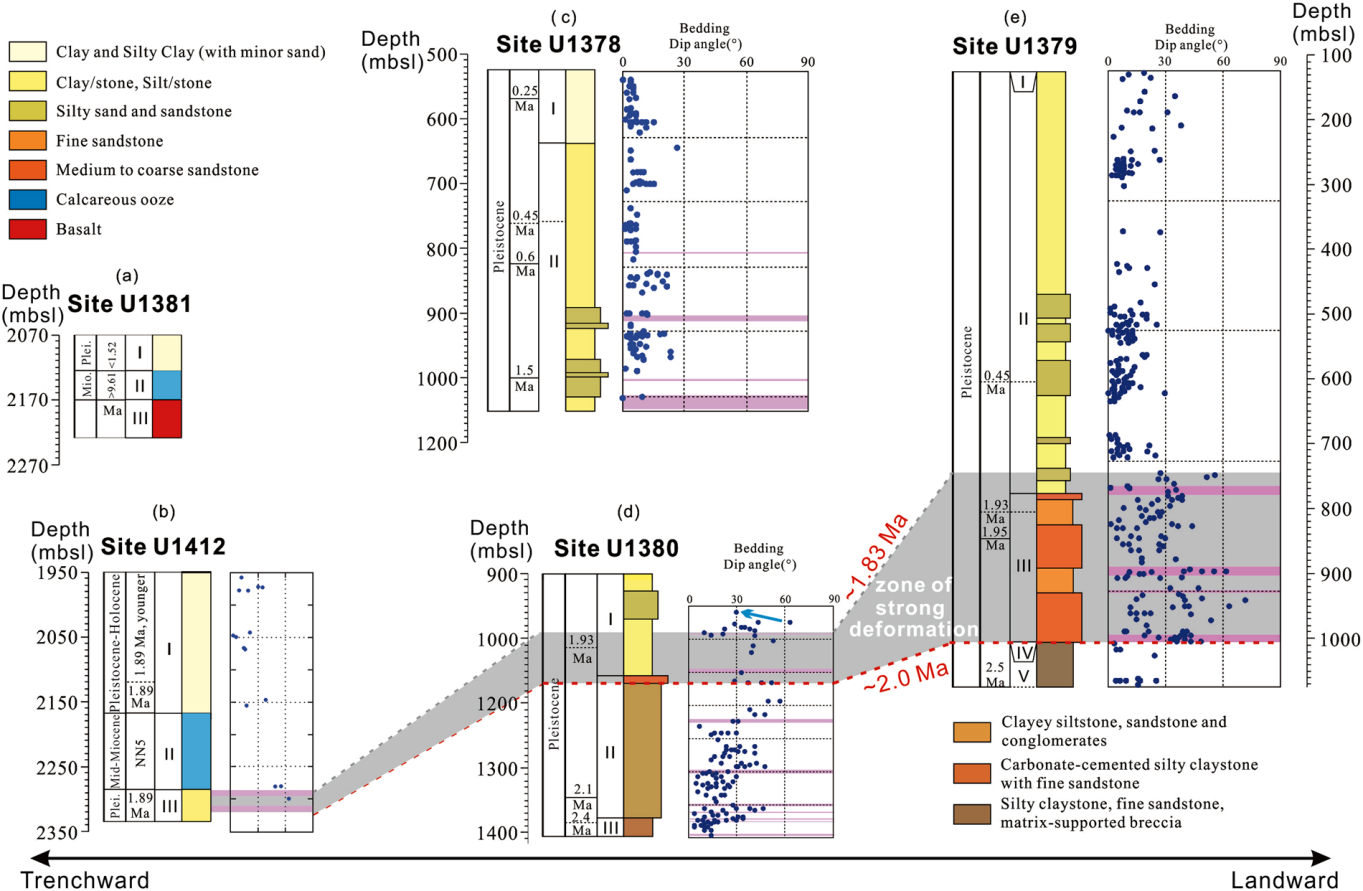
Integrated stratigraphy of IODP Expedition 334 and 344 Sites U1381, U1412, U1378, U1380, and U1379 (modified after^8,32^). (**a**–**e**) Show the chronology, major lithologic units, and bedding dip angles of these sites. The shaded grey zone marks the relatively strong deformation zone recognized at Sites U1412 (**b**), U1380 (**d**, Fig. 6) and U1379 (**e**). The sediments of this zone spans ~2.0 to ~1.83 Ma. The relatively strong deformation likely occurred briefly at ~1.80 Ma, i.e., immediately after the rapid forearc subsidence associated with the dramatic subduction erosion by the subducting Cocos Ridge (also see text). Purple bands indicate fracture zones. Depth is in meters below sea level (mbsl).

The magnetostratigraphic results show that the zone of relatively strong deformation occurs within the Olduvai subchron and can thus be constrained to ~1.94 to ~1.83 Ma by linear interpolation (Fig. 5). The shell fragment-bearing coarse sandstone unit underlying the relatively strong deformation zone is dated at ~1.95 Ma (Fig. 5). The timing of deformation can be bracketed by ages of the youngest deformed strata and the oldest non-deformed strata. Therefore, the deformation must have occurred after ~1.83 Ma. The upper bound of the deformation zone occurs in the lower part of Unit I that consists mainly of silty clay. The lithology of Unit I of Site U1380 is the same as that of Unit II of Site U1378, which is situated slightly trenchward in the middle slope (Figs. 1b, 7c, 7d). The strata at Site U1378 are gently dipping, mostly 10°–20° or less (Fig. 7c), suggesting that these strata were not deformed. The basal age of non-deformed Unit II of Site U1378 is slightly older than 1.5 Ma based on the biostratigraphic data^8^ (Fig. 7c). Therefore, the strong deformation in the middle slope must have occurred prior to ~1.5 Ma. The timing of deformation may be further refined. The strata immediately above the relatively strong deformation zone at Site U1380 display generally steep dipping, but appear to show a shift toward shallow dipping (<30°) upsection (Fig. 7d, the blue arrow). The shift occurs at ~470 mbsf, which is near the upper boundary of the Olduvai subchron at ~1.78 Ma (Fig. 5). As such, the relatively strong deformation can be further constrained to prior to ~1.78 Ma. Taken together, the relatively strong deformation at the middle slope of the forearc likely occurred between ~1.83 Ma and ~1.78 Ma, i.e., probably briefly at ~1.80 Ma.

To investigate whether the strong deformation is widespread in the forearc, we examine the lithological and structural data of the IODP sites drilled from the frontal prism (Site U1412) and the upper slope (Site U1379) of the forearc (Fig. 1b). At upper slope Site U1379, the interval between ~760 and ~1030 meter below sea level (mbsl) (or ~630 and ~880 mbsf), exhibits more abundant faults, fractures, and steeper beds (dip angles >30°) than other depths (Fig. 7e), clearly indicating a zone of relatively strong deformation. The strong deformation zone is also underlain by a shelf facies unit, Unit IV, that was dated at 2.2 ± 0.2 Ma^7^. The upper bound of this zone occurs in the lowermost part of Unit II and its age may be constrained by the nearest two biostratigraphic datums of 1.93 Ma (~680 mbsf) and 1.95 Ma (~720 mbsf) in the upper part of the relatively strong deformation zone^7^ (Fig. 7e). Assuming a constant sedimentation rate for the upper part of this zone, the age of the upper bound is estimated to be ~1.91 Ma. Therefore, the deformation must have occurred after ~1.91 Ma. The lack of definitive biostratigraphic datum immediately above the upper bound in the non-deformed strata prevents the minimum age of deformation from being precisely determined. The occurrence of nannofossil *Helicosphaera selli* about 10 m above the upper bound indicates an age of >1.47 Ma^8^, which is compatible with the < 1.91 Ma deformation. This zone of relatively strong deformation at Site U1379 correlates well with that at Site U1380. Both zones occur above the shell fragment–bearing shelf facies. Also, both zones display comparable age intervals, spanning around ~2.0 to ~1.87 Ma. In addition, the occurrence of the relatively strong deformation zones in different lithologies of similar age intervals at both Site U1380 and Site U1379 (Fig. 7d, 7e) supports the notion that the strong deformation occurred briefly, likely at ~1.80 Ma.

At frontal prism Site U1412, abundant faults, fractures, and foliations occur in Unit III at Site U1412 (~330 to 370 mbsf)^8^, indicating a zone of relatively strong deformation. Also, the foliations are largely shallower than the bedding within this interval^8^, suggesting strong sub-horizontal compressional deformation at the frontal prism site. The upper bound age of the strong deformation zone is Pleistocene, around 1.89 Ma based on the biostratigraphic data^8^, which is similar to the estimated upper bound ages of the strong deformation zones at Sites U1380 (~1.83 Ma) and U1379 (~1.91 Ma), respectively.

Collectively, these data indicate an interval of sediments spanning ~2.0 to ~1.83 Ma across the forearc that likely underwent a wholesale brief deformation at ~1.80 Ma. Also, the intensity of deformation appears to decrease from the trench to the upper slope according to the depicted deformation model (Fig. 6c). The occurrence of abundant fissility/foliations that are shallower than the bedding in the deformation zones at Sites U1412 and U1380 may suggest intense deformation in the frontal prism and the middle slope, whereas the occurrence of faults, fractures, and steep strata, but without abundant fissility/foliations^8^ in the relatively strong deformation zone at Site U1379 may indicate relatively moderate deformation in the upper slope. On the other hand, lithological difference may also contribute to the structural difference across the forearc. Nevertheless, the deformation of the stratigraphically coeval sediments is significant. The shell fragment-bearing sandstones underlying the relatively strong deformation zones mark shelf facies deposited in the uplifted forearc upon the arrival of the CR at the MAT^7^. The overlying deep-water facies that occupies the deformation zones represents deposition during the subsequent rapid forearc subsidence^7^ driven by the sudden, effective, and dramatic subduction erosion of the CR. In this context, the strong brief deformation of the deep-water facies at ~1.80 Ma across the forearc is best explained by the intense sub-horizontal shear immediately following the rapid forearc subsidence. Therefore, the widespread, brief, and relatively strong deformation is likely an integral part of the consequences in response to the sudden, dramatic subduction erosion associated with the near-flat subduction of the CR at the Costa Rican margin. Since subduction erosion causes the landward migration of the trench and subsidence in the forearc area in an erosive convergent margin, similar styles of deformation are probably common in other erosive convergent margins as well. The findings thus contribute to improved understanding of the crustal material transfer, hydrothermal fluid migration, and seismogenesis in erosive convergent margins.

## Methods

A total of 353 standard cylindrical paleomagnetic specimens were drilled from the working halves of sediment cores retrieved from IODP Expedition 344 Hole U1380C. Among these specimens, 60 were selected for shipboard studies, which included stepwise alternating field (AF) or thermal demagnetization up to 120 mT and 475 °C, respectively, to isolate the characteristic remanent magnetization (ChRM). The remaining specimens were investigated on shore. Most specimens from the upper part of the hole disintegrated during shipment, only 193 specimens arrived intact. Their low-field magnetic susceptibility (MS) and anisotropy of magnetic susceptibility (AMS) were first measured with an AGICO Kappabridge KLY-3. About half of these specimens were subsequently subjected to stepwise AF demagnetization up to 50 to 100 mT at increments of 5 or 10 mT using a Molspin demagnetizer. The other half of the specimens were thermally demagnetized using an ASC TD-48 thermal demagnetizer at increments of 30° to 50 °C, typically in 20 steps, up to 600 °C. Magnetic remanence was measured with a 2G Enterprises Inc. 755 superconducting rock magnetometer housed in a magnetically shielded room (residual field <300 nT) in the Paleomagnetism Laboratory of Nanjing University, China.

The demagnetization data were presented graphically using vector endpoint plots^44^ and were analyzed using principal component analysis^45^. The software PuffinPlot^46^ and PaleoMac^47^ were used to analyze the data and produce figures.

The AMS, bedding dip, and ChRM data are summarized in Tables S1 and S2 in the supplementary information.

### Data availability statement

The data are provided in the Supplementary Information.

## Electronic supplementary material

Supplementary Information
