# Supplementary material for: Paleomagnetism of IODP Site U1380: Implications for the Forearc Deformation in the Costa Rican Erosive Convergent Margin
Source: Sci Rep. 2018 Jul 30;8:11430. doi: 10.1038/s41598-018-29243-7 (PMC6065392; doi:10.1038/s41598-018-29243-7)
Supplement: Supplementary file 1 — Supplementary Information [file 41598_2018_29243_MOESM1_ESM.pdf]

Supplementary Information for

Paleomagnetism of IODP Site U1380: Implications for the Forearc Deformation in the Costa Rican Erosive Convergent Margin

Yong-Xiang Li[1]*, Xixi Zhao[2]*, Siyi Xie[1], Luigi Jovane[3], Katerina Petronotis[4]

[1] State Key Laboratory for Mineral Deposits Research, School of Earth Sciences and Engineering, Institute of Geophysics and Geodynamics, Nanjing University, Nanjing 210046, China
[2] State Key Laboratory of Marine Geology, Tongji University, Shanghai 200092, China
[3] Instituto Oceanográfico, Universidade de São Paulo, São Paulo SP 05508, Brazil
[4] International Ocean Discovery Program, Texas A&M University, College Station, Texas 77845, USA

Content

Table S1 Comparison of K_{min} tilt and the measured dip of bedding
Table S2 Summary of the paleomagnetic data of Hole U1380C (ChRMs)

*Email: yxli@nju.edu.cn; or xzhao@tongji.edu.cn

Table S1 Comparison of Kmin tilt and the measured dip of bedding

Sample ID	Depth (m)	Kmin tilt	Depth (m)	Bedding Dip
3R5-16	453.86	60	459.45	30
5R3-11	470.21	28	473.55	44
6R1-49	477.29	30	474.57	63
6R1-100	477.8	34	477.24	29
6R1-126	479.56	36	481.96	35
6R2-130	479.6	27	482.54	33
6R3-35	480.15	30	484.38	38
6R3-86	480.66	37	486.42	22
6R3-107	480.87	23	487.3	41
6R4-36	481.66	30	489.61	10
6R6-18	484.26	32	491.53	21
6R6-78	484.84	28	493.26	14
6R7-88	486.68	22	494.16	41
7R2-43	488.43	30	502.3	52
7R4-47	491.47	26	510.55	40
8R1-11	496.31	18	518.86	39
8R1-69	496.89	30	550.3	33
8R3-3	499.23	32	564.26	37
8R3-70	499.9	31	564.38	29
8R4-67	501.37	29	564.91	52
8R4-99	501.69	33	565.19	45
8R5-7	502.27	36	593.25	57
8R5-69	502.89	37	593.6	49
9R3-59	509.49	50	605.2	38
10R3-11	517.36	43	612.87	41
12R5-62	541.62	36	613.67	42
15R1-100	565.1	49	613.76	47
16R1-57	574.37	41	623.3	30
17R2-9	585.09	45	623.42	29
19R1-52	603.52	43	632.76	7
19R1-83	603.83	38	642.39	17
19R2-18	604.68	44	642.4	17
19R3-24	606.24	32	642.41	16
20R1-14	612.84	34	642.66	20
20R2-89	615.09	56	642.7	25
22R2-26	624.16	25	642.76	30
22R2-109	624.99	17	642.85	29
23R1-17	641.97	27	643.45	14
23R1-91	642.71	23	651.68	18
23R2-5	643.35	25	662.1	32
23R2-71	644.01	18	662.57	41
23R2-92	644.22	25	663.33	23
24R1-4	651.54	24	666.47	27
24R1-66	652.16	34	667.72	31
25R1-12	661.32	15	667.85	35
25R2-25	661.45	35	667.92	29
25R2-73	663.43	25	667.99	24
25R2-104	663.74	21	668.21	30
25Rcc-8	664.28	25	671.47	42
26R1-47	666.47	13	673	22
26R2-7	667.57	20	676.1	16

26R2-74	668.24	28	676.62	22
27R1-74	671.64	25	677.05	21
27R2-129	673.69	25	677.74	26
28R1-42	676.12	20	681.36	14
28R1-88	676.58	21	685.93	43
28R2-24	677.44	20	686.46	20
28R2-60	677.8	79	686.93	33
28R2-80	678	25	687.27	32
30R2-72	687.62	13	690.69	31
31R1-124	691.68	18	690.75	38
33R1-138	701.38	13	690.91	30
33R2-37	701.87	16	691.41	47
33R2-94	702.44	17	691.85	36
33R3-26	703.26	11	700.76	24
34R1-106	705.86	11	701.23	13
35R2-30	709.6	14	701.62	9
35R2-75	710.05	15	701.97	11
35R3-24	711.33	9	702.67	17
36R2-98	716.98	13	704.98	15
36R2-108	717.08	16	706.8	30
36R3-10	717.6	14	707.08	6
36R3-34	717.84	14	707.32	20
37R2-132	721.67	20	710.42	17
37R3-6	722.36	11	715.13	21
38R1-62	724.72	20	716.1	28
38R1-76	724.86	19	717.15	5
38R3-44	727.54	19	717.88	12
39R1-87	729.87	21	719.5	17
39R2-98	731.48	21	719.77	24
40R1-90	734.8	13	720.08	9
40R1-105	734.95	16	721.79	25
41R1-26	739.06	19	721.96	17
41R1-113	739.93	25	722.16	14
42R1-14	743.84	11	724.57	22
42R2-24	745.44	10	725.86	13
42R3-26	746.96	26	730.44	6
44R1-34	753.84	48	734.36	11
44R2-50	755.5	45	734.76	20
45R3-43	761.73	21	735.34	22
46R1-15	763.35	21	739.87	16
46R1-131	764.51	23	745.77	17
46R2-19	764.89	25	751.9	27
47R1-34	768.34	22	753.74	38
47R1-71	768.71	41	754.73	10
48R1-48	773.38	15	755.78	31
48R2-88	775.28	16	756.14	46
48R3-45	776.35	7	758.86	22
49R1-119	778.89	18	759.45	41
49R2-6	779.26	13	760.51	15
49R2-60	779.8	13	762.74	4
50R1-9	782.69	13	763.52	24
50R1-110	783.7	23	764.25	18
50R2-4	784.14	14	764.8	15
50R2-97	784.91	7	765.01	18

50R3-4	785.64	14	768.21	10
50R3-63	786.23	13	769.27	34
51R1-8	787.58	15	769.43	30
51R1-23	787.73	14	770.23	28
51R2-28	788.48	6	770.54	34
51R2-71	789.71	10	773.08	30
52R1-37	794.17	13	773.43	17
52R1-60	794.4	19	773.49	6
52R1-124	795.04	21	774	8
52R3-27	797.07	26	774.51	23
			775.11	16
			775.29	18
			776.02	11
			777.91	15
			778.39	9
			780.2	8
			783.13	4
			784.3	4
			784.62	3
			785.14	10
			785.26	11
			787.57	15
			787.87	14
			788.52	10
			794.7	14

Note: Kmin tilt, 90-Kmin inclination; selected proximal depth levels of the two data sets are highlighted in yellow for the ease of comparison of Kmin tilt of AMS data and the measured bedding dip.

Table S2 Summary of the paleomagnetic data of Hole U1380C (ChRMs)

Sample ID	Depth (m)	Dg (°)	Ig(°)	MAD	Kmin_Az of Dip	Kmin_Dip	Ds (°)	Is (°)
2R1-55	438.55	67.5	-23.5	15.8				
3R2-64	449.84	159.6	5.4	9.7				
3R4-63	452.57	276.8	-40.8	12.3				
3R5-16	453.86	231.8	62.1	15.5	130	60	162.6	31.7
4R3-32	459.52	231	22.4	9				
5R2-39	468.66	160.7	0.6	11.2				
5R3-11	470.21	196.8	11.3	13.9	188	28	197	-16.4
5R4-94	471.61	96.4	10.4	12.4				
5R5-14	471.88	1.6	1.4	9.8				
5R5-51	472.25	270.5	28	9.3				
5R7-41	474.25	244.7	29.6	6.5				
6R1-49	477.29	77	18.6	15.3	131	30	80.9	-0.1
6R1-100	477.8	2.6	-4.3	5	121	34	4.6	11.7
6R1-126	479.56	351.1	-2.4	5.6	122	36	355.7	20.5
6R2-130	479.6	307.9	8.7	15.8	301	27	308.2	-18.1
6R3-35	480.15	244.3	38.2	13.5	293	30	255.1	16
6R3-86	480.66	29.2	50.1	6.4	68	37	329.2	66
6R3-107	480.87	18.4	14.1	6.8	47	23	13	33.8
6R4-36	481.66	17.9	4	4.6	47	30	13	29.8
6R6-18	484.26	258.5	36.9	14.8	308	32	269.3	13.5
6R6-37	484.45	295.1	3.5	7.4				
6R6-78	484.84	230.7	36.3	15.4	263	28	236.9	11.7
6R7-6	485.64	3.4	52	9.1				
6R7-88	486.68	18.6	53	13.5	376	22	17.8	31
7R2-43	488.43	83.8	35.8	14.1	161	30	100.5	24.6
7R4-47	491.47	256.4	13.9	15.2	273	26	256.6	-11.1
7R4-108	492.08	176.3	11.3	10.4				
7R6-42	493.82	332.3	-7.9	10.4				
8R1-11	496.31	87	-1.7	11.8	114	18	85.6	-17.7
8R1-69	496.89	50.2	49.6	15.4	35	30	81.6	76.5
8R1-121	497.41	139.4	47.4	6.5				
8R3-3	499.23	107.4	47.9	12.9	145	32	119.1	20.3
8R3-70	499.9	121.2	58.9	7.5	143	31	130.3	29.1
8R4-67	501.37	121.8	20.9	14.1	141	29	123	-6.6
8R4-99	501.69	73.3	29.3	14.7	149	33	86.9	17
8R5-7	502.27	4.4	47.5	10.8	67	36	320.1	51.2
8R5-69	502.89	223.5	68.7	14.8	223	37	223.2	31.7
9R2-66	507.88	56.8	-16.9	9.5				
9R3-59	509.49	344.5	58.2	12.2	303	50	324.1	14.1
9R5-39	511.57	22.6	40.5	2.4				
10R3-11	517.36	217.4	67.7	10.2	184	43	197.6	27.4
10R3-136	518.61	49.8	44.7	15.3				
10R5-131	520.84	243.2	24	8.1				
11R5-85	530.39	127.2	26.1	15.5				
12R3-58	538.21	120.5	23.4	13.2				
12R5-62	541.62	12.4	57.6	11.7	83	36	318.1	52
13R1-29	544.99	233	42.9	11.8				
13R3-7	547.29	157.6	40.3	7.7				
13R5-45	551.15	239.6	56.4	11.9				
15R1-100	565.1	337.9	-3.8	5.2	237	49	337.1	5.7
16R1-57	574.37	8	-5	14.3	263	41	7.7	5.9

17R2-9	585.09	232.9	27.3	6.1	161	45	219.4	7.4
18R1-6	593.26	294.5	18.8	3.5				
19R1-52	603.52	21.8	9.9	14.3	298	43	16.7	3
19R1-83	603.83	332.6	29.8	5.9	249	38	315.1	19.4
19R2-18	604.68	183.1	-7.9	13.2	54	44	168.7	-32.2
19R3-8	605.07	13.6	13.7	14				
19R3-24	606.24	215.9	-15.1	13.4	89	32	203.6	-31.9
20R1-14	612.84	279.8	2.9	12.1	160	34	273.9	18.6
20R2-89	615.09	69.5	14.3	12.8	325	56	50.8	19.8
21R1-101	623.41	322.4	-10.6	2				
22R2-26	624.16	179.2	2.3	4.1	83	25	179.6	-0.5
22R2-109	624.99	174.1	6.5	2.3	92	17	172.6	3.9
22R2-124	633.91	9.2	5.1	2.8				
23R1-17	641.97	311.6	14.7	2.3	194	27	302.3	25.4
23R1-91	642.71	45.5	13	11.8	317	23	40.5	11.4
23R1-115	642.95	215.3	20.4	7.5				
23R2-5	643.35	103.9	17.2	15	16	25	111.6	16.4
23R2-71	644.01	281.6	15.8	3.5	192	18	276.6	14.9
23R2-92	644.22	138.5	25.3	10.6	55	25	150.6	25.5
23R2-112	644.29	317.3	14	7.7				
24R1-4	651.54	110.5	-1.6	5.2	357	24	109.3	7.9
24R1-66	652.16	10	1.9	5	274	34	7.9	4.9
25R1-12	661.32	310.1	45.5	7.8	182	15	294.9	53.2
25R2-25	661.45	81.5	29.4	6.6	321	35	57.1	41
25R2-55	662.63	33.7	20.9	12.1				
25R2-73	663.43	96.9	31.8	10.4	349	25	80	36
25R2-104	663.74	41.1	1.7	7.3	323	21	41.3	-2.6
25Rcc-8	664.28	225.7	0.8	2.6	97	25	222.7	16.1
26R1-47	666.47	351.8	-4.5	8.4	247	13	352.4	-1.1
26R1-61	666.61	344.3	51.8	8.2				
26R2-7	667.57	156.7	0.4	9.4	37	20	155.3	-9.4
26R2-38	667.88	253.9	-3.8	3.7				
26R2-74	668.24	200	10.8	3.1	90	28	192.7	18.9
27R1-48	671.38	258.8	-17.3	14.2				
27R1-74	671.64	115.9	-11.1	13.9	347	25	117	4.9
27R2-97	673.37	115.3	-43.2	6.2				
27R2-129	673.69	312.4	-6.1	4.3	183	25	311.8	9.8
28R1-42	676.12	28.7	17.6	9.5	264	20	21.4	28
28R1-61	676.31	156.2	-22.6	4.9				
28R1-88	676.58	359.7	4.6	7.5	204	21	357.4	23.6
28R2-24	677.44	73.1	21.4	4.9	9	20	81.9	28.8
28R2-51	677.71	2.4	11.9	5.7				
28R2-60	677.8	156.6	31.4	15.7	261	79	200.7	17.9
28R2-80	678	356.4	24.2	11.3	270	25	346.1	20.3
30R1-27	685.67	244.3	39	9.6				
30R2-72	687.62	259.7	-40	9	124	13	265.8	-30.2
31R1-36	690.8	144.8	31.6	8.5				
31R1-124	691.68	82.6	42.5	5.3	35	18	99.1	52.8
32R2-23	696.1	93.9	19.4	10.5				
33R1-138	701.38	30.9	27.9	8.5	325	13	25.5	22
33R2-37	701.87	84.5	47.8	11.8	355	16	67.7	45.3
33R2-94	702.44	220.4	45.6	13.4	185	17	213.3	31.1
33R3-8	702.63	118.6	12.5	8.6				
33R3-26	703.26	115.8	4	8.6	3	11	116.1	-0.3

34R1-106	705.86	100.3	27	15.5	23	11	106.1	28.9
34R2-83.5	706.965	297.1	10.1	8				
34R2-128	707.58	6.8	17.5	8.3				
35R2-30	709.6	212	-18.1	7	70	14	208.1	-28.9
35R2-75	710.05	312.5	8.4	5.1	213	15	310	10.6
35R3-24	711.33	322.9	9.4	13	229	9	321.4	9.9
35R3-73	711.82	103.7	45.2	3.3				
36R2-98	716.98	347.1	15.8	4.2	265	13	343.7	13.6
36R2-108	717.08	252.3	11.9	9.5	142	16	248.4	17
36R3-10	717.6	318	25.9	8.8	251	14	312.7	19.8
36R3-34	717.84	134.9	27	7.8	67	14	142.4	31.4
36R3-58	718.08	210.8	24.9	4.9				
37R1-76	720.06	298.1	11.1	5.8				
37R2-132	721.67	142.6	11.2	2.7	87	20	147.7	21.8
37R2-141	721.76	148.6	6.8	8.6				
37R3-6	722.36	29.1	16.4	10.4	11	11	30.5	26.8
38R1-45	724.55	302.7	10.7	7.8				
38R1-62	724.72	334.5	16.4	7.1	249	20	329.1	13.9
38R1-76	724.86	143.8	30.5	7	73	19	155.8	34.9
38R1-138	725.48	344	38.6	8.2				
38R2-40	726	284.7	12.7	11.6				
38R2-71	726.31	279.9	16.1	11.7				
38R3-31	727.41	270.1	18	6				
38R3-44	727.54	276.6	13	9.9	233	19	275.2	-1
38RCC－5	728.75	37.9	9.6	8.7				
39R1-24	729.24	235.3	9.9	6.1				
39R1-48	729.48	73.1	2.8	9.3				
39R1-63	729.63	140	21.4	8.6				
39R1-87	729.87	69.1	10.5	13.9	35	21	73.4	27.5
39R2-16	730.66	64.4	2.6	8.6				
39R2-98	731.48	312.5	21.6	10.9	267	21	308.9	6.3
40R1-39	734.29	168	38.1	5				
40R1-90	734.8	189.2	13.8	14.5	93	13	185.9	14.8
40R1-105	734.95	190.7	19.3	13.7	100	16	185.2	18.7
41R1-26	739.06	281.4	-19.8	7.8	188	19	287.8	-17.6
41R1-113	739.93	161.6	18.7	5.8	99	25	156.8	6.1
41R2-13	740.43	151	52.6	7.3				
42R1-14	743.84	106.2	26.6	13.2	5	11	111.3	24
42R2-24	745.44	294.2	14.8	10.8	299	10	294.3	4.8
42R3-26	746.96	334.3	31	4.6	338	26	334.8	5
43R2-38	750.23	34.9	42.5	3.5				
43R2-64	750.74	209.3	44.1	5.9				
43R2-93	751.03	102.5	47.7	9.2				
44R1-34	753.84	263.5	44.3	7.5	283	48	269.2	-1.9
44R1-76	754.26	353.8	40.4	4.9				
44R2-50	755.5	164.9	56.8	8.9	166	45	165.4	11.8
44R2-73	755.73	324.7	41.5	6.6				
44R3-62	757.12	233.8	37.3	13.9				
45R1-13	758.43	280.7	19.5	12.9				
45R2-18	759.31	346.9	23.8	8.1				
45R2-59	759.72	223.2	23.3	8.6				
45R3-8	761.38	196.8	20.9	2.9				
45R3-43	761.73	108.1	11.7	6.9	37	21	113.4	17.6
45R5-24	762.3	312.6	14.7	3				

45R5-55	762.61	209.8	19.5	11.3				
46R1-15	763.35	337.5	11.7	8.2	241	21	332.8	13.2
46R1-131	764.51	280.3	14.5	7.9	196	23	275.1	11.1
46R2-4	764.71	215	0.8	8.7				
46R2-19	764.89	214.8	-9.2	8.3	139	25	220	-14.3
47R1-34	768.34	63.3	17.7	6.5	328	22	56	18.4
47R1-71	768.71	28.4	40.3	12.6	358	41	20.7	3.2
47R1-129	769.29	189.6	57.6	3				
48R1-48	773.38	283.2	20.6	7.7	194	15	277.7	19.7
48R2-10	774.4	133.8	19.3	2.8				
48R2-44	774.84	16.6	36.2	2.4				
48R2-88	775.28	156.9	24.7	3.1	62	16	163.8	22.4
48R3-45	776.35	198.2	11.2	8.6	134	7	197.1	8.1
49R1-30	778	159.8	11.4	6.9				
49R1-119	778.89	59.4	17.2	4.1	16	18	65.1	29.7
49R2-6	779.26	82	17.4	2.5	27	13	86.2	24.5
49R2-60	779.8	192.9	7.8	1.6	111	13	191.4	5.8
49R2-89	780.05	272	-1.1	4				
50R1-9	782.69	222.1	18.3	6	147	13	218.4	14.5
50R1-67	783.27	337.1	7.6	8.1				
50R1-110	783.7	57.9	7.5	5.2	339	23	55.9	2.6
50R2-4	784.14	190.6	15.4	7	123	14	187.7	9.7
50R2-54	784.48	236.2	20.2	4.2				
50R2-97	784.91	280.1	28.4	5.6	235	7	277.7	23.4
50R3-4	785.64	217.1	24.6	5.3	173	14	213.8	14.2
50RCC-7	785.9	166.5	27.6	6.2				
50R3-63	786.23	277.4	20.8	4.6	217	13	273.9	14
51R1-8	787.58	111.6	37	3.4	64	15	122.3	46.1
51R1-23	787.73	95.8	38.5	6.9	62	14	104.2	49.6
51R2-28	788.48	21.5	24.9	4.4	343	6	20	20.2
51R2-66	788.86	293	-33.7	2				
51R2-71	789.71	69	22.1	2.8	92	10	70.2	12.9
52R1-37	794.17	184.7	29.8	5.6	127	13	179.5	22.3
52R1-60	794.4	149.4	19.5	5.1	93	19	145.5	8.4
52R1-76	794.56	144.6	25.5	3.4				
52R1-124	795.04	352.1	22.6	13.2	290	21	346.5	11.8
52R3-27	797.07	185.3	19.6	15.4	137	26	181.7	1.5

Note: Dg, Ig= Declination, Inclination of ChRM defined by the principal component analysis (PCA); MAD, maximum angular deviation; Kmin_Az of Dip, 180 +Declination of Kmin axis; Kmin_dip, 90-Inclination of Kmin axis; Ds, Is=Declination, Inclination of ChRM after tilt correction The samples that do not have the Kmin data, i.e., AMS data, include those that were demagnetized on the IODP drillship Jodies Resolution (JR) and do not have perfect shape such that measurements of their AMS were deemed unreliable.
